# Glycogen as an advantageous polymer carrier in cancer theranostics: Straightforward *in vivo* evidence

**DOI:** 10.1038/s41598-020-67277-y

**Published:** 2020-06-26

**Authors:** Andrea Gálisová, Markéta Jirátová, Mariia Rabyk, Eva Sticová, Milan Hájek, Martin Hrubý, Daniel Jirák

**Affiliations:** 1 MR Unit, Department of Radiodiagnostic and Interventional Radiology, Institute for Clinical and Experimental Medicine, Prague, Czech Republic; 2 Department of Physiology, Faculty of Science, Charles University, Prague, Czech Republic; 3 Institute of Macromolecular Chemistry, Academy of Sciences of the Czech Republic, Prague, Czech Republic; 4 Department of Clinical and Transplant Pathology, Institute for Clinical and Experimental Medicine, Prague, Czech Republic; 5 Department of Pathology, Third Faculty of Medicine, Charles University, Prague, Czech Republic; 6 Institute of Biophysics and Informatics, First Faculty of Medicine, Charles University in Prague, Prague, Czech Republic; 7 Department of Science and Research, Faculty of Health Studies, Technical University of Liberec, Liberec, Czech Republic

**Keywords:** Drug delivery, Nanoparticles, Cancer imaging

## Abstract

As a natural polysaccharide polymer, glycogen possesses suitable properties for use as a nanoparticle carrier in cancer theranostics. Not only it is inherently biocompatible, it can also be easily chemically modified with various moieties. Synthetic glycogen conjugates can passively accumulate in tumours due to enhanced permeability of tumour vessels and limited lymphatic drainage (the EPR effect). For this study, we developed and examined a glycogen-based carrier containing a gadolinium chelate and near-infrared fluorescent dye. Our aim was to monitor biodistribution and accumulation in tumour-bearing rats using magnetic resonance and fluorescence imaging. Our data clearly show that these conjugates possess suitable imaging and tumour-targeting properties, and are safe under both *in vitro* and *in vivo* conditions. Additional modification of glycogen polymers with poly(2-alkyl-2-oxazolines) led to a reduction in the elimination rate and lower uptake in internal organs (lower whole-body background: 45% and 27% lower MRI signals of oxazoline-based conjugates in the liver and kidneys, respectively compared to the unmodified version). Our results highlight the potential of multimodal glycogen-based nanopolymers as a carrier for drug delivery systems in tumour diagnosis and treatment.

## Introduction

The majority of anticancer drugs are highly toxic and adversely affect healthy cells due to a lack of specific targeting^1,2^. Therefore, a safe drug delivery system capable of accumulating and providing controlled drug release at the tumour site is highly desirable. Nanoparticles represent a versatile carrier for drug delivery due to their ability to passively accumulate in solid tumours. This occurs via the enhanced permeability and retention (EPR) effect, a relatively universal phenomenon whereby nano-sized structures (up to approx. 200 nm in size) become entrapped in tumour tissue due to either leaky endothelia or aberrant tumour vessel architecture. Restricted or even completely absent lymphatic drainage in tumours limits the release and passive accumulation of compounds^3^. Nanotherapeutic carrier can accumulate in tumours via the EPR effect without any targeting ligand^4–6^. Their size above the renal threshold (molecular weight > ca 45 kDa/hydrodynamic diameter > 8 nm) leads to prolonged circulation in the bloodstream increasing the probability of accumulation at the target site^3^. Importantly, polymers can control the release of drugs, being composed of a structure responsive to external stimuli, such as pH changes in acidic cancer-cell environments^7^, the presence of enzymes, and redox potential^8,9^. Thus to minimise the drug release in the bloodstream and the cytotoxic effect on healthy cells^10–12^.

One limitation of nanoparticles is that they tend to opsonise, clearing quickly through the reticuloendothelial system in the liver and spleen^13^. However, this can be mitigated by surface modification: for instance, grafting nanoparticles with poly(ethylene oxide) (PEO) (known also as poly(ethylene glycol) – PEG) is a well-established procedure for reducing nanoparticle toxicity, prolonging circulation in the bloodstream, and minimising interactions with the immune system^14^. On the other hand, PEO also has the potential to be both immunogenic and antigenic and degrading to produce reactive oxygen species^15,16^. Therefore, alternative polymers such as poly(2-oxazolines) (POx) are currently the subject of extensive testing^15,17,18^. POx are significantly more stable compared to PEO^19,20^, retain their non-fouling (protein-repellent) potential, have low unspecific organ deposition and do not exhibit immunogenicity^15,21,22^. This so-called “stealth” effect leads to the prolonged circulation of coated nanoparticles in the blood and increases the likelihood of accumulation in tumour tissue via the EPR effect^22^.

In addition to other tried-and-tested drug delivery systems such as proteins^23^, synthetic polymers^24–27^ and carbon nanotubes^28^, natural carbohydrate polymers such as polysaccharides are versatile and unique carriers due to their high biocompatibility, biodegradability, and availability^29–32^. Polysaccharides can also be easily modified to control drug release while incorporating both diagnostic and therapeutic entities. Polysaccharide surfaces can also be tailored with ligands for more specific targeted delivery, e.g. using folate to bind to overexpressed folate receptors on cancer cells^33^.

Most studies of polysaccharide drug delivery systems use alginate^34^, dextran^35,36^, chitosan^37,38^, pectin^39–42^, starch^43^ or hyaluronic acid^44,45^. However, only a few studies have explored the potential of glycogen^32,46^. Glycogen is a natural hyperbranched polymer of suitable size for the EPR effect (hydrodynamic diameter ≈ 50 nm, molecular weight ≈ 10 MDa) and above the renal threshold, it is degraded into D-glucose by intracellular enzymes and further metabolized by normal physiological glycolysis^46^. Moreover, it is biocompatible, biodegradable^47^, widely available as a natural renewable resource (oysters, plants)^48,49^, and can be easily modified with drugs or diagnostic moieties, what makes glycogen ideally suited for use in the construction of a multimodal drug delivery system^32,46,50^.

In the absence of evidence to indicate glycogen is efficient as a nanocarrier in a clinically relevant model such as a tumour-bearing animal, we performed a detailed characterisation of a modified glycogen carrier under *in vivo* conditions on an animal tumor model of hepatocellular carcinoma (HUH7 human cancer cell line). Glycogen was modified with two imaging moieties: a gadolinium chelate for magnetic resonance (MR) and a near-infrared fluorescent dye for fluorescence imaging. A multimodal agent was deemed most favourable given each method provides a different set of information on biodistribution, accumulation, and clearance of probes under *in vivo* conditions. In order to enhance tumour-targeting properties and eliminate any possible adverse effects on other organs, the polymers were modified with POx. Using tumour-bearing rats, we evaluated imaging properties, biodistribution, safety, *in vivo* accumulation, and clearance. To the best of our knowledge, this is the first demonstration of imaging potential of these contrast agents on *in vivo* animal model.

## Results

### Imaging properties of polymers

**Figure 1:**
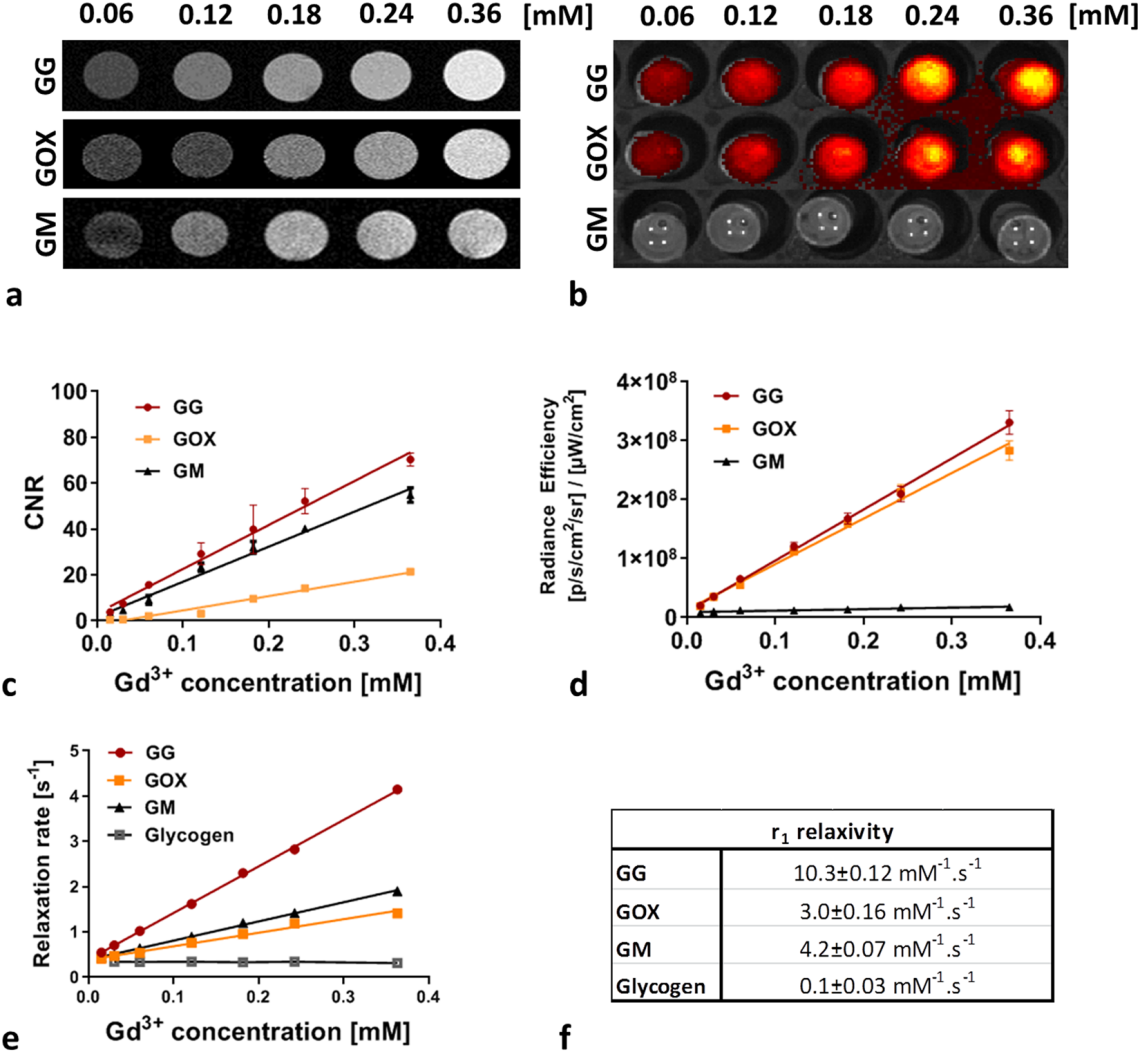
Characteristics of glycogen-based conjugates: (**a**) MR and (**b**) fluorescence images of conjugates at different Gd^3+^ concentrations, (**c**) with corresponding contrast-to-noise ratios (CNR) and (**d**) fluorescence radiance efficiency values; (**e**) dependence of MR relaxation rates R_1_ on Gd^3+^ concentration of probes and (**f**) the corresponding r_1_ relaxivities measured at 0.5 T.

Two polymer conjugate variants were synthetized: glycogen-based conjugates modified with POx (GOX) and glycogen-based conjugates without POx (GG). A commercially available MR contrast agent gadoterate meglumine (GM) served as a control. MR properties of the conjugates were examined by MR imaging at 4.7 T and relaxometry at 0.5 T. T_1_-weighted MR signals and corresponding contrast-to-noise ratio (CNR) values were linearly dependent on concentration of Gd^3+^ in the conjugates (Fig. 1a,c), with the highest signal issuing from GG. Relaxivity of the GG probe (10.3 ± 0.12 mM^−1^s^−1^) was higher compared to the GM (4.2 ± 0.07 mM^−1^s^−1^) and GOX (3.0 ± 0.16 mM^−1^s^−1^) probes (Fig. 1e,f). Relaxivity of unmodified glycogen without imaging agents and oxazolines was 0.1 ± 0.03 mM^−1^s^−1^ (Fig. 1e,f). The higher CNR values of GG and GOX compared to GM reflect higher r_1_ relaxivities. Fluorescence emission of probes was measured after specific excitation at 745 nm. While conjugates containing the fluorescent dye emitted a strong fluorescence signal linearly dependent on probe concentration, the control sample (GM) emitted no fluorescence signal after excitation (Fig. 1b,d). The fluorescence signals of GG and GOX were similar.

### **MR and fluorescence*in vivo*imaging of rats administered with conjugates**

**Figure 2:**
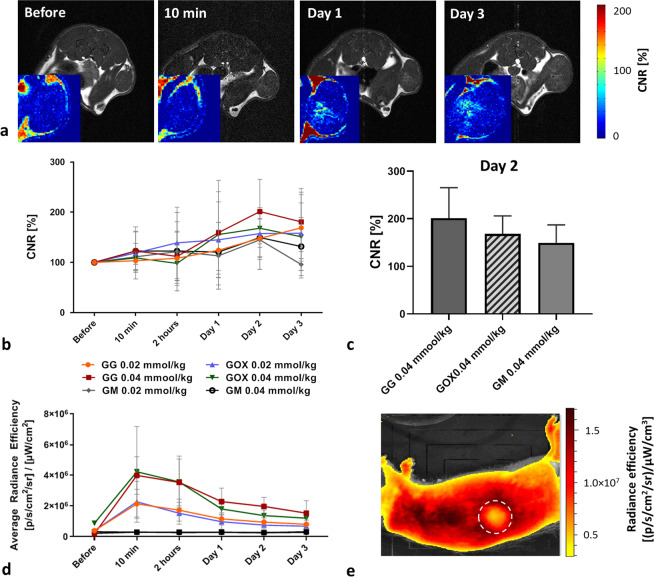
Accumulation of glycogen-based conjugates in tumours: (**a**) T_1_-weighted images of rats injected with GG; corresponding CNR maps (insets) show the accumulation of probes in tumour centres; (**b**) quantification of MR signals from tumours before and after probe injection; (**c**) CNR values in tumours on day 2 show higher accumulation of GG compared to GOX and GM; (**d**) fluorescence signals originating from tumours at different time points after glycogen probe injection; (**e**) representative fluorescence image of a rat injected with GG (sagittal plane); dotted circle marks the tumour area.

After intravenous administration of conjugates, we assessed biodistribution and accumulation of the glycogen-based probes (GG and GOX) and control agent (GM) using MR and fluorescence *in vivo* imaging (Fig. 2). The MR imaging experiment revealed accumulation of the glycogen-based conjugates in the tumour tissue with a higher uptake of GG and GOX agents compared to GM (Fig. 2b). The highest CNR values were obtained from tumours injected with GG throughout the whole examination (Fig. 2b,c). The GOX signal was lower than GG but higher than GM (Fig. 2c). The highest content of the probes in tumours was found on day 2 (Fig. 2b,c); the CNR values in tumours on day 2 reflecting accumulation of probes are shown in Fig. 2c. Accumulation of GG of concentration 0.02 mmol/kg increased between day 2 and 3, however this increase was within the standard deviation and not statistically significant. With the administration of higher probe concentrations, MR signals in tumours increased (Fig. 2b). The signals of the control sample (GM) was lower than GG and GOX throughout the whole examination confirming the target properties of the glycogen-based conjugates toward the tumour tissue. A CNR map analysis revealed increased MR signals, predominantly in tumour centres (Fig. 2a). To confirm that MR signals increased in line with accumulation in glycogen-based conjugates and not in fatty tissue (which gives also a positive signal on T_1_-weighted MR images), we carried out histological analysis of tumour tissues. Histology excluded the presence of fibrosis and steatosis, only necrosis was found in tumour centres.

Accumulation of GG and GOX in tumour tissues was confirmed also by fluorescence *in vivo* imaging (Fig. 2e). Fluorescence signals of all probes rapidly declined on day 1 (Fig. 2d) and therefore fluorescence is not suitable for assessing the long-term changes of probe accumulation. GG and GOX at 0.04 mmol Gd^3+^/kg concentration levels remained above pre-injection values even 3 days after injection.

**Figure 3:**
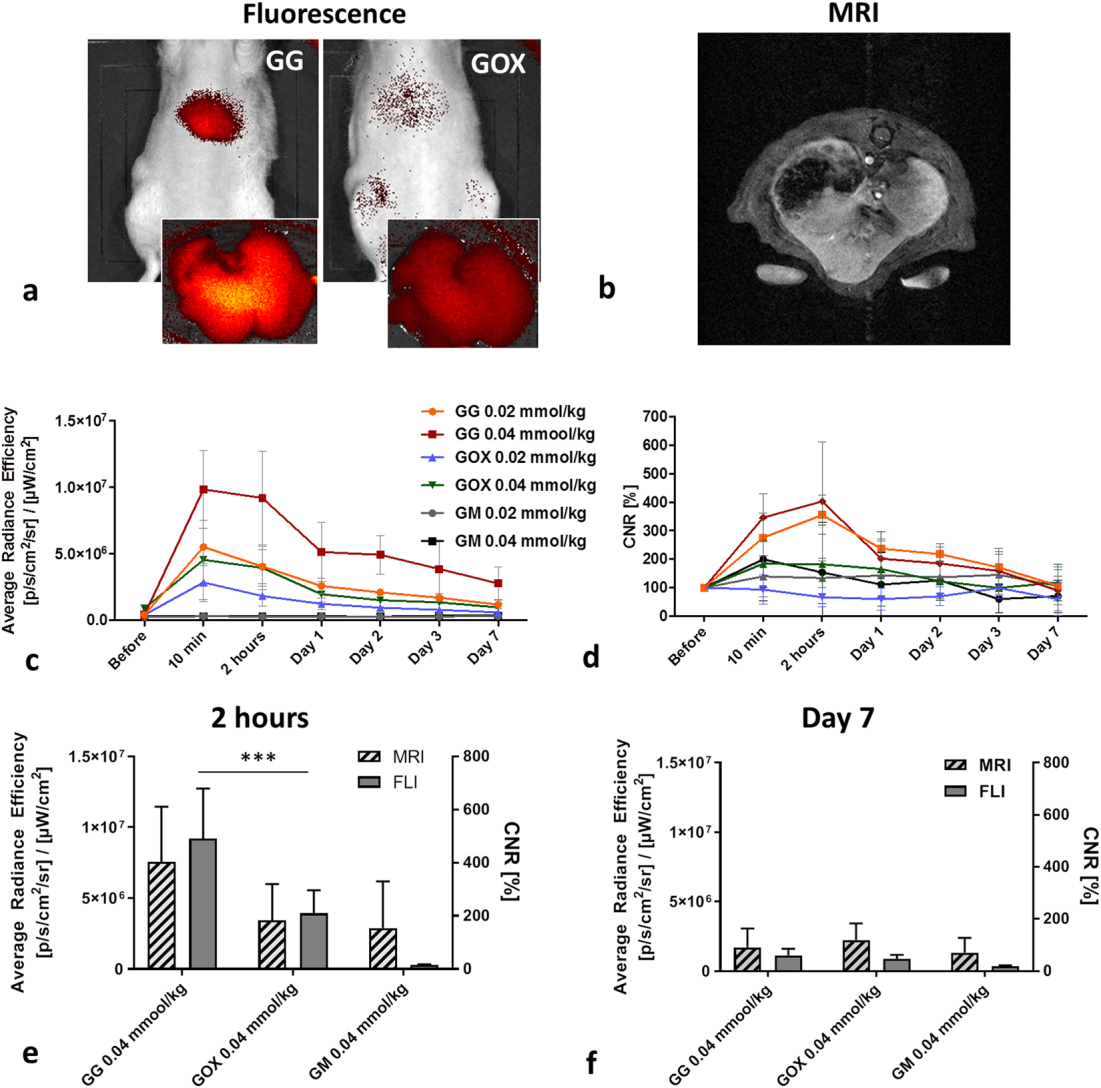
Accumulation of glycogen-based conjugates in the liver: (**a**) Representative fluorescence and (**b**) MRI images of rat livers injected with glycogen-based probes, (**a**) with insets showing detailed *ex vivo* fluorescent liver images; (**c**) quantification of fluorescence and (**d**) MRI signals from rat livers at different time points after probe administration; fluorescence (FLI) and MRI signals (**e**) 2 hours and (**f**) 7 days after injection reveal differences in the highest probe concentrations (0.04 mmol Gd^3+^/kg).

The probes tested were also accumulated in liver tissue. Higher MRI and fluorescence signals were observed in rat livers injected with GG compared to GOX throughout the whole examination (p < 0.05, paired t-test) (Fig. 3). The highest uptake was observed 2 hours after intravenous administration, with GG accumulation significantly higher than GOX (p = 0.0001) (Fig. 3f). MRI signals of all probes decreased to pre-injection values on day 7, despite the presence of a slightly elevated fluorescence of GG in the liver (Fig. 3d). Both MRI and fluorescence signals showed a similar trend in the liver (Fig. 3e,f). The accumulation of the commercially-available probe (GM) was observed only within the first post-injection day.

**Figure 4:**
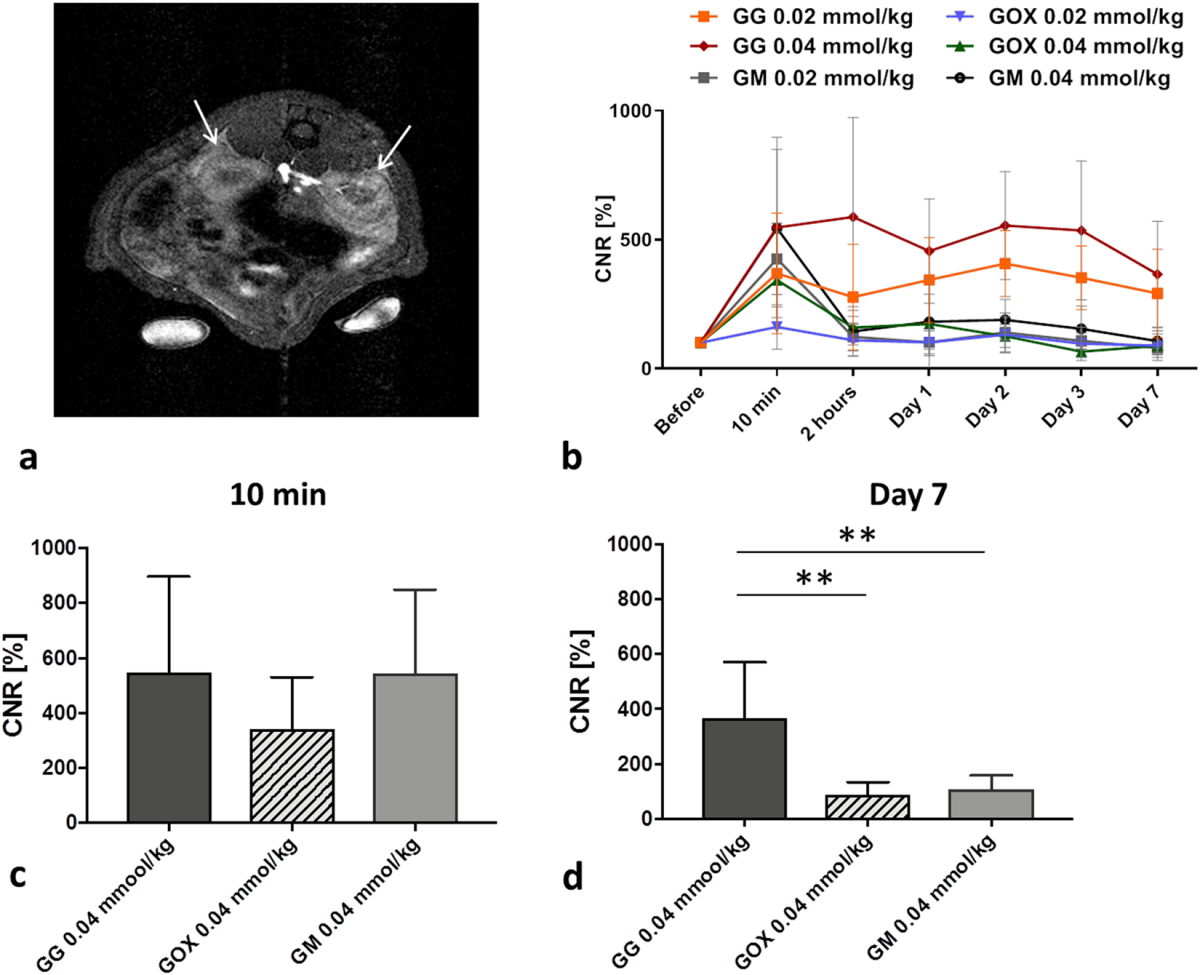
Accumulation of glycogen-based conjugates in the kidneys: (**a**) Representative MR image of kidneys (marked with arrows) after GG administration; (**b**) CNR values over time after glycogen-based probe administration; (**c**) quantification of CNR values originating from the highest probe concentrations (0.04 mmol Gd^3+^/kg) at two different time points – (**c**) 10 min and (**d**) day 7.

Due to the attenuation of fluorescence in deeper tissues, probe uptake in the kidneys was analysed by MRI only. Signals from the GM control and modified glycogen-based GOX conjugate decreased to initial values 2 h after injection. The GG signal increased above the pre-injection value throughout the whole examination even at the lowest probe concentration (Fig. 4b) (p < 0.01, paired ANOVA test; p < 0.01 between GG and GOX, GG and GM on day 7, unpaired ANOVA) (Fig. 4d). The signal from the control probe GM was observed immediately after injection only and then it was cleared out.

### ***Ex vivo*fluorescence imaging**

Fluorescence imaging is limited by its attenuation and refraction of optical signals in biological tissues, particularly in deeper structures. Therefore, to assess probe accumulation more precisely and to confirm the results of *in vivo* imaging, *ex vivo* fluorescence analysis of the internal organs (liver, kidneys, spleen and tumours) was performed at two time points: day 2 and day 7.

**Figure 5:**
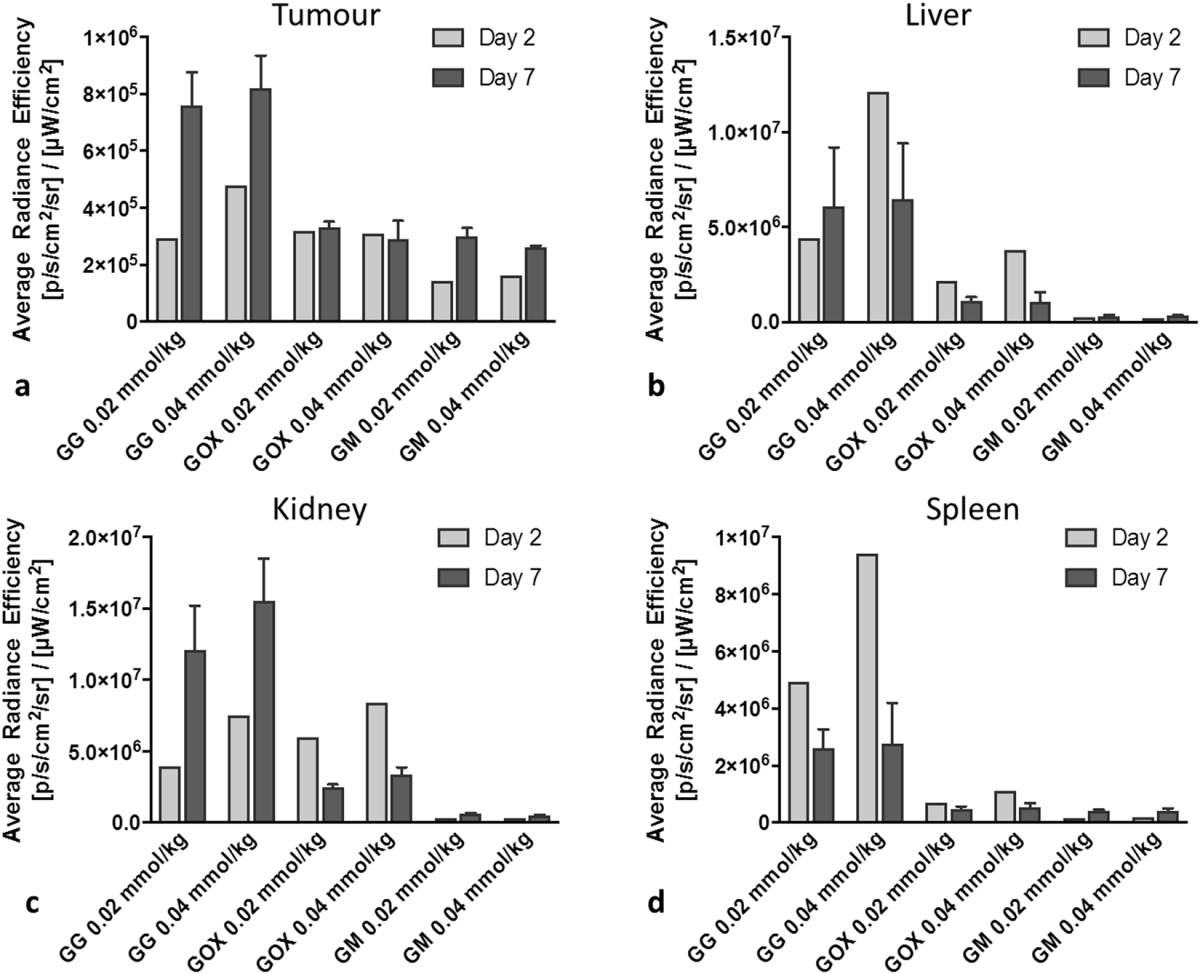
Quantification of *ex vivo* fluorescence signals in (**a**) tumours, (**b**) livers, (**c**) kidneys, and (**d**) spleens; fluorescence signals were higher on day 7 in tumours and kidneys.

Corresponding to our *in vivo* MRI data, *ex vivo* analysis revealed GG continuously accumulated in tumours, with the highest uptake on day 7 following probe injection (Fig. 5a). GOX accumulation on day 2 was lower than GG, again in line with our *in vivo* imaging results. GOX shows relatively the same accumulation in the tumours on day 2 and day 7 in contrast to GG, which was uptaken faster on day 2. Fluorescence signal values from GOX and GM were within background signal ranges.

Apart from the lowest GG probe concentration, probe uptake in the liver was higher on day 2 than on day 7, a finding that corresponds with our *in vivo* results (Fig. 5b).

Similar to *in vivo* MRI, *ex vivo* fluorescence analysis of the kidneys confirmed higher accumulation of GG than GOX on day 7 (Fig. 5c) with a decrease in accumulation from day 2 to day 7. There was an initial accumulation of GG in the spleen on day 2, but no uptake of the other agents (GOX, GM). A persistent low fluorescence GG signal was also present in the spleen on day 7 (Fig. 5c). This discrepancy between the MRI and FLI signals from the kidneys on day 2 could be explained by lower r_1_ relaxivity of the GOX conjugate and thus lower T_1_ signal on the T_1_-weighted MR images. Nevertheless, the fluorescence signals of GOX and GG on day 2 obtained by the *ex vivo* fluorescence are very similar and not statistically different.

Overall, probe accumulation increased over time in the tumours and kidneys. However, probe signals diminished over time after initial uptake in the liver and spleen.

### Toxicity and biocompatibility of conjugates

MTT assay revealed no signs of toxicity in the conjugates tested. The level of absorbance at 570 nm reflected the level of cell proliferation. Compared to non-treated control cells, HUH7 cells incubated with glycogen-based polymers showed comparable or slightly higher absorbance (see Supplementary Table S1 online).

Serum levels of ALT, bilirubin, creatinine, and albumin were within physiological range, reflecting the healthy status of the polymer-injected rats. AST levels slightly increased after agent administration (including GM). However, it should be noted that control rats without tumours also exhibited higher AST values. Therefore, the increase might be connected to the specific rat strain used. Importantly, no difference was found between biochemical levels of all measured compounds in rats injected with glycogen-based probes and in those injected with commercial GM probes (see Supplementary Fig. S1 online).

**Figure 6:**
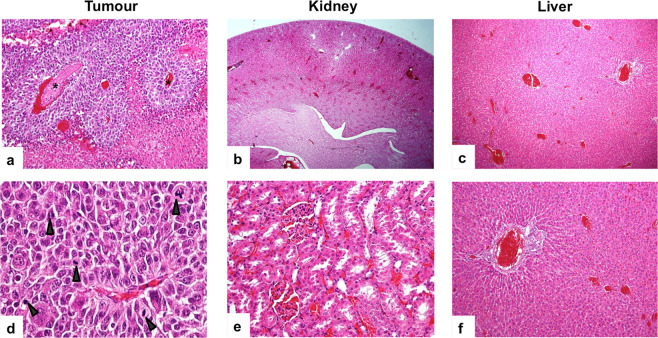
Histology of tumor and parenchymal organs after administration of the glycogen-based conjugates. (**a**) Tissue sections stained with haematoxylin-eosin (H&E) show tumor tissue with confluent coagulation necrosis (magnification 400×). (**d**) Neoplastic cells display significant nuclear atypia with increased mitotic activity (arrows) (magnification 600×). (**b**,**e**) H&E staining shows renal parenchyma (b, magnification 20×; e, magnification 400×) and (**c**,**d**) liver tissue (c, magnification 40×; f, magnification 100×) of preserved architecture with no apparent pathological changes after administration of the glycogen-based probes.

Histological analysis confirmed the absence of significant pathological changes in parenchymal organs after administration of glycogen-based conjugates (Fig. 6). There were no necroinflammatory changes, steatosis or fibrosis in the liver tissue.

## Discussion

Glycogen nanoparticles represent a versatile, biocompatible, and biodegradable platform for polymer-based carriers, possessing advantageous physicochemical properties for biomedical applications^32^. They can also be modified with imaging probes, allowing them to be tracked using non-invasive imaging methods. In this study, we tested glycogen-based conjugates bearing imaging entities for MR visualisation and optical imaging in rats with solid tumours. Our aim was to monitor under experimental conditions *in vivo* behaviour of a glycogen-based carrier suitable for a wide variety of imaging methods and biological applications. Our polymers were also modified with bioconfluent POx to increase conjugate accumulation in tumour tissue, thus prolonging biodegradation and blood circulation time.

To prove the visualisation ability and sensitivity of probes using MR and optical imaging methods, we examined solutions of different conjugate concentrations. MR imaging, MR relaxometry, and fluorescence imaging proved suitably sensitive to glycogen nanoparticles. Boasting high MR relaxivity, good contrast on T_1_-weighted MR images, and strong fluorescence signals, the nanoparticles enable multimodal *in vivo* tracking. Even at the lowest tested concentrations, probe signals were clearly detectable (0.06 mM of Gd^3+^) on MR images. Notably, glycogen concentrations of synthesised nanoparticles can even be increased where required. Although POx can enhance tumour targeting by slowing biodegradation and blood circulation time, our POx-modified polymers exhibited lower MR relaxivity, decreasing imaging effectiveness as a result. This effect may have been due either to the more restricted availability of water molecules in gadolinium chelates, or to the higher conformational mobility of gadolinium chelates at the end of POx chains compared to chelates directly attached to the glycogen core.

To evaluate drug delivery, polymer probes were tested for biodistribution, accumulation, and clearance in hepatocellular xenograft tumours using an animal cancer model. Our *in vivo* multimodal imaging results clearly confirmed the accumulation of glycogen-based conjugates in tumour tissue, displaying sustained MR contrast enhancement. Contrast in glycogen-based conjugates was higher than that observed in the commercial MR contrast agent, the plausible explanation being continuous accumulation due to the EPR effect. The difference between the commercial GM agent and the glycogen-based conjugates was highest on day 3 after i.v. injection. Glycogen-based probe accumulation peaked on day 2 in the case of GG (concentration of 0.04 mmol Gd^3+^/kg), confirming it the carrier most suitable for drug delivery to tumours. It should be noted that tumours were monitored only until day 7, at which point massive tumour necrosis occurred (confirmed by histology), a finding that obviously compromises the validity of our MR data. Probe accumulation in tumours was also confirmed by *in vivo* and *ex vivo* fluorescence imaging. We observed a rapid decline in the *in vivo* fluorescence signal within the first days after administration. Previous animal model studies to have used other imaging probes report the dissociation of MR and fluorescence signals, a similar effect possibly caused by lysosomal degradation of fluorescent dyes^51,52^.

Our *in vivo* MR data show that throughout the entire examination POx conjugates issued the lower MR signals, a result more attributable to lower MR relaxivity (3.4-times lower compared to GG) than to lower accumulation in tumours considering the corresponding fluorescence signals revealed no significant differences between the probes with and without POx. However, non-POx probes exhibited slightly higher fluorescence signals (for both *in vivo* and *ex vivo*), accumulating in greater amounts as a result.

All drug delivery systems need to minimise the toxic effect of the drug incorporated while mitigating effects on non-targeted tissue^1,2^. To that end, the use of beneficial non-biofouling (protein-repellent) compounds such as POx and PEO^14^ is recommended. Confirming the stealth effect of POx, our model demonstrates that conjugates modified with POx slowed the elimination rate and reduced the uptake of POx-modified conjugates in rat livers, spleens and kidneys compared to the unmodified version (lower body background ratio; 45% and 27% lower MRI signals of oxazoline-based conjugates in the liver and kidneys, respectively compared to the unmodified version). We observed significantly lower *in vivo* MRI and fluorescence signals from POx-modified conjugates in the liver and kidneys throughout the whole examination, a finding supported by our *ex vivo* analysis of fluorescence signals from internal organs. Based on our results comparing tumours with other organs, POx not only caused internal organs to be protected against probe uptake but, in contrast to our expectations, inhibited accumulation in target tumour tissue.

The merits of any drug delivery system are largely dependent on its ability to ensure efficient clearance from the body. In our study, both MR and fluorescence imaging revealed that probes were eliminated from the organism mainly through the kidneys, similar to the preferred elimination route of MR chelates such as DOTA. As predicted, polymer modification with oxazolines reduced renal clearance. Elevated MR signals in the kidneys of rats administered polymers without POx persisted even 7 days after application, raising safety concerns and underscoring the importance of oxazoline modification. However, it should be noted that Gd^3+^ probe concentrations were much lower (up to 0.04 mmol Gd^3+^/kg) compared to standard doses routinely administered in human medicine (approximately 0.1 mmol Gd^3+^/kg)^53^. Therefore, we assume any adverse effect of Gd^3+^ was minimal. Moreover, in our model, we found no pathological changes in organs, confirmed by histological and biochemical analysis of blood proteins. Given our glycogen-based conjugates were uptaken by cells via endocytosis and intracellularly degraded into D-glucose units^46^, we assume they degraded to form water and carbon dioxide in tumour tissue. Similar to commercial clinically approved Gd-based MR contrast agents, we hypothesise the released chelate (DOTA) and fluorescent dye were then cleared from the body by the kidneys or liver. Nevertheless, it should be noted that these conjugates can carry other imaging moieties, thus impacting on sensitive radionuclides and certain applications such as ^19^F MR probes.

The most important factor to consider when designing any drug delivery system is toxicity risk. Our biochemistry and immunohistochemistry results confirm the non-toxicity and biocompatibility of the glycogen-based carriers tested. Incubated cells exposed to probes showed comparable viability and proliferation to controls. Moreover, no prominent changes in blood serum protein levels were detected after intravenous probe administration in rats. Importantly, histology examination revealed no pathological changes in internal organs (liver, kidneys or spleen) after administration.

A key advantage of our drug delivery model for future applications is that it can be tailored to release drugs at particular sites of interest and/or under specific biological conditions, e.g. pH changes in solid tumours^54^, thus providing safe and effective anticancer treatment for specific types of tumours.

## Conclusion

In this study, we demonstrate that our novel glycogen-based compounds are suitable for multimodal imaging of solid tumours, being of the requisite biological properties for tumour targeting. Biocompatible and safe, these compounds can be easily modified to control the biological fates of these conjugates. Although oxazoline modification prevents uptake of probes in internal organs, it also inhibits accumulation in target tumour tissue. Both our *in vitro* and *in vivo* results highlight the potential of glycogen-based probes as carriers for drug delivery systems in tumour diagnosis and treatment.

## Methods

Glycogen (type II from oysters) was used for nanoparticle synthesis. Two polymer conjugate variants were prepared: glycogen-based conjugates modified with POx (GOX) and glycogen-based conjugates without POx (GG). Glycogen conjugates grafted and non-grafted with GOX were synthesised as previously described^46^.

### MR imaging and relaxometry of polymers

MR imaging was performed on a 4.7 T scanner (Bruker BioSpin, Germany) using a resonator coil with an internal diameter of 7 cm (Bruker BioSpin, Germany). MR images were processed using ImageJ software (version 1.46r, National Institute of Health, USA). All optical images (fluorescence) were acquired on an IVIS Lumina XR imager (Perkin Elmer, USA) and processed using Living Image software (Perkin Elmer, USA).

We examined the MR and optical properties of GG, GOX, and GM, a commercial MR contrast agent used as a control. MR relaxivity (r_1_) of conjugates was assessed by measuring T_1_ relaxation times at various probe concentrations (0.01–0.36 mM Gd^3+^) on a 0.5 T relaxometer (Bruker BioSpin, Germany) using a saturation recovery sequence (recycle delay 12 s or 5 s depending on T_1_ values).

T_1_-weighted MR images of conjugates in tubes were acquired using a Rapid Acquisition with Refocused Echoes (RARE) sequence (repetition time (TR) = 125 ms, echo time (TE) – 11.6 ms, spatial resolution 0.2 × 0.2 × 1.5 mm^3^, scan time 6 min 24 s). A tube filled with water served as a reference. Regions of interest (ROI) of the same size were drawn around each sample in MR images, with the CNR between the reference and sample of choice calculated for each agent concentration.

Fluorescence images of the same samples were acquired during a 2-second exposure using aperture (f/stop) 4 and binning 4. Fluorescence excitation was set at 745 nm and emission at 810–875 nm. ROIs were drawn around each tube, with the emitted optical signal expressed as the radiance efficiency ([photons/sec/cm^2^/sr]/(μW/cm^2^)). Fluorescence images were overlaid on photographs to localise the optical signal.

### Animal model

All animal protocols were approved by the Ethics Committee of the Institute for Clinical and Experimental Medicine and the Ministry of Health of the Czech Republic (No. 58/2014) in accordance with the European Communities Council Directive (2010/63/EU). Animals were kept in ventilated cages under a 12-h light cycle and given free access to food and water.

Tumours were induced in immunodeficient RNU nude rats (Velaz, Czech Republic) by subcutaneous injection of HUH7 cells (5 × 10^6^) above the right hind leg. During implantation, animals were anaesthetised by isoflurane inhalation (5% for induction, 1% for maintenance). Prior to implantation, cells were cultured for 2 weeks in a DMEM culture medium supplemented with 10% foetal bovine serum (FBS), 5% L-glutamine, and a 5% penicillin/streptomycin solution (37 °C, 5% CO_2_).

Animals (n = 48) were divided into 6 experimental groups according to the type of conjugate administered and based on two concentrations: GG 0.02 mmol Gd^3+^/kg; GG 0.04 mmol Gd^3+^/kg; GOX 0.02 mmol Gd^3+^/kg; GOX 0.04 mmol Gd^3+^/kg; GM 0.02 mmol Gd^3+^/kg and GM 0.04 mmol Gd^3+^/kg (n = 8 in each group).

### *In vivo* examination – MR and fluorescence imaging

Animals were kept under general inhalation anaesthesia for the duration of all *in vivo* imaging experiments (isoflurane: 5% for induction, 1% for maintenance). Body temperature was maintained using a heating system, with breathing monitored throughout.

Three weeks after tumour induction, T_1_-weighted MR images of tumours, livers and kidneys were acquired before and after intravenous administration of GG, GOX and GM at two concentrations (0.02/0.04 mmol Gd^3+^/kg). MR imaging was performed before, immediately after, and then 10 min, 2 h, 1, 2, 3 and 7 days after conjugate administration.

MRI examination parameters were as follows: spin-echo sequence for tumour imaging with TR = 125 ms, TE = 11.6 ms, spatial resolution 0.25 × 0.25 × 1.50 mm^3^, 8 acquisitions, scan time 4 min 16 s; gradient echo sequence for the liver and kidneys: TR = 72 ms, TE = 4.6 ms, spatial resolution 0.25 × 0.25 × 1.50 mm^3^, 12 acquisitions, scan time 3 min 41 s. CNR values of the tumour, liver and kidneys in relation to muscle tissue were calculated at each time point from ROIs manually outlined around each tissue type. CNR maps were calculated based on pixel-wise processing of MR images using a custom-written script in Matlab (Matlab, MathWorks, Natick, MA, USA).

After MRI, near-infrared fluorescence images (excitation at 745 nm, emission at 810–875 nm) of rat tumours and livers were acquired based on a 30-second exposure time, aperture 4 (f/stop) and binning 4. Fluorescence images were overlaid on photographs for anatomic localisation of the optical signal. The average fluorescence efficiency ([photons/sec/cm^2^/sr]/(μW/cm^2^)) was calculated from each ROI outlined around the liver and tumour tissues.

Toxicity and biocompatibility was evaluated with the MTT test and biochemical analysis of the blood serum proteins. Details of the tests are listed in the Supplementary Information.

One week after intravenous administration of the probes, selected rats were sacrificed (n = 2 per each group) and internal organs removed for histological analysis. Tumours, livers, kidneys and spleens were placed in a 4% formaldehyde solution overnight (pH 7.4) at 4 °C and then embedded in paraffin blocks. Paraffin samples were cut into 4-μm tissue sections and routinely stained with haematoxylin and eosin. Each organ was examined for the presence of pathological changes by an experienced pathologist.

### Statistical analysis

Statistical analysis was performed using GraphPad Prism 6.02 (GraphPad Prism Software Inc, USA). A standard two-tailed Student’s t-test was used to compare differences between groups. Comparisons of three or more groups were performed by analysis of variance (one-way ANOVA), with the significance level set at p < 0.05. For mean values and standard deviations, see graphs.

## Supplementary information

Supplementary information.

## Data Availability

All relevant data are included in the manuscript and the supplementary information. Used datasets are available from the corresponding author on reasonable request.
