# Supplementary material for: Glycogen as an advantageous polymer carrier in cancer theranostics: Straightforward in vivo evidence
Source: Sci Rep. 2020 Jun 26;10:10411. doi: 10.1038/s41598-020-67277-y (PMC7320016; doi:10.1038/s41598-020-67277-y)
Supplement: Supplementary file 1 — Supplementary information. [file 41598_2020_67277_MOESM1_ESM.pdf]

Supplementary Information

Glycogen as an advantageous polymer carrier in cancer theranostics: Straightforward in vivo evidence

Andrea Gálisová[1], Markéta Jirátová[1,2], Mariia Rabyk[3], Eva Sticová[4,5], Milan Hájek[1], Martin Hrubý[3], Daniel Jirák[1,6,7*]

Toxicity and biocompatibility evaluation

To assess probe toxicity, HUH7 cells (cell density 0.1 × 10^5 /ml) were seeded in a 96-well plate and, after 72 hours, incubated for 24 hours with GG and GOX (Gd^{3+} concentration 0.014; 0.072; 0.36 and 1.8 mmol/l) in tetraplicate. Cells were subsequently washed with HBSS, and 200 µl of fresh media added to each well. After 3 days, the medium was removed, with 250 µl of the MTT solution (5 mg/mL in RPMI 1640; Sigma Aldrich, Czech Republic) added over a 6-hour period. The MTT solution was then discarded from the wells followed by the addition of 200 µl per well of dimethyl sulfoxide (DMSO; Sigma Aldrich, Czech Republic) and 30 µl of glycine buffer. Absorbance (at 570 nm) was measured immediately on the Multi-Mode Microplate Reader (Synergy™, BioTek®Instruments, Inc, USA).

MTT Absorbance at 570 nm			
**Concentration [mM]**	**GG**	**GOX**	**Control**
0.4	1.60±0.16	1.48±0.36	1.75±0.50
0.07	1.71±0.11	1.79±0.31	
0.014	1.55±0.26	2.14±0.70	
0.0028	2.14±0.53	2.26±1.08	

Table S1. MTT toxicity test results; absorbance measured at 570 nm confirmed non-toxicity of the glycogen-based conjugates.

To evaluate the biocompatibility of probes, blood samples were collected from all rats using a catheter administered into the tail vein at specific time points: before and after application of the conjugates on days 1, 3 and 7. The blood sample was left to clot at room temperature for 20 min, with the sample then centrifuged (6000 rpm, 10 minutes). The resulting serum was then removed and stored at -20 ºC until examination. Concentrations of alanine aminotransferase (ALT), aspartate aminotransferase (AST), bilirubin, creatinine, and albumin were assessed using the DRI-CHEM 500 analyser (Fujifilm, Japan).

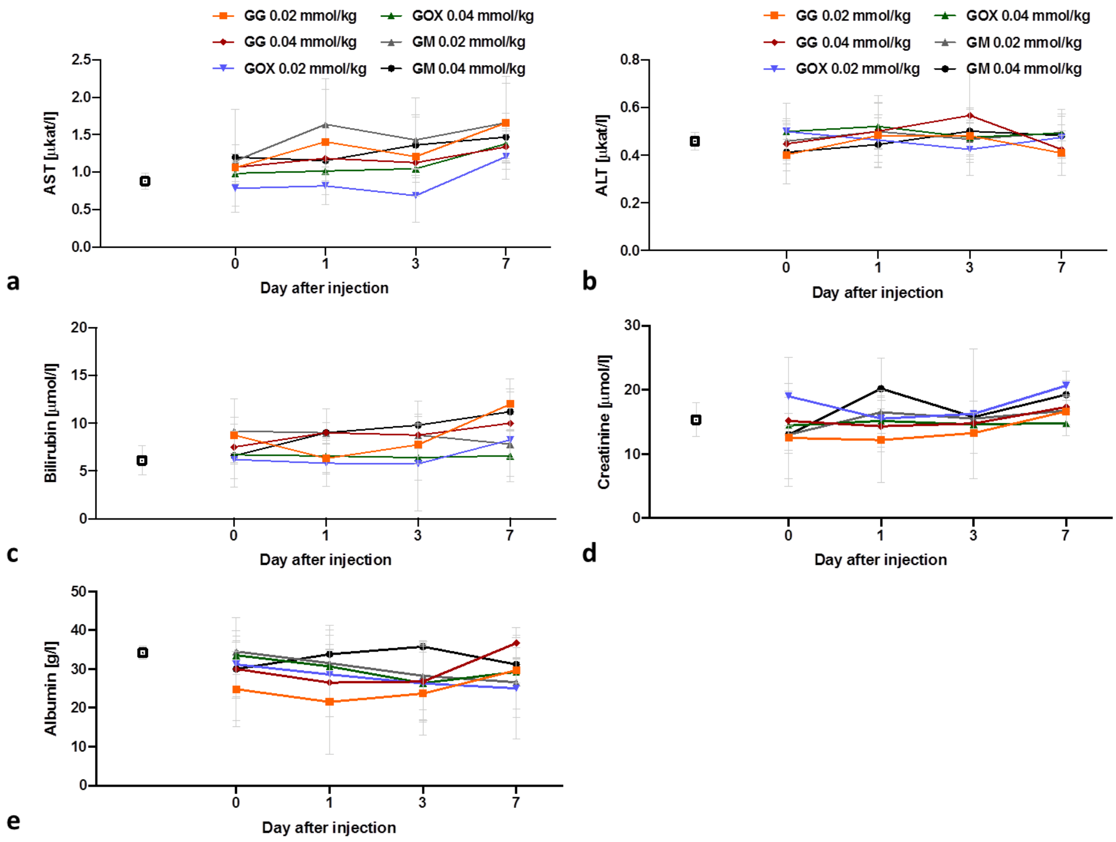

Figure. S1. Changes to biochemical compounds in blood serum after administration of glycogen-based conjugates: Levels of (a) aspartate aminotransferase (AST), (b) alanine aminotransferase (ALT), (c) bilirubin, (d) creatinine, and (e) albumin in the blood serum of rats at different time points after i.v. administration of glycogen-based conjugates at different concentrations; squares in graphs represent the basal values of compounds in the blood of rats without induced tumours.
